# Supplementary material for: Prevalence and risk factors of abuse against older adult women: A cross-sectional community study in Eastern Andalusia, Spain
Source: PLoS One. 2026 May 15;21(5):e0349515. doi: 10.1371/journal.pone.0349515 (PMC13178880; doi:10.1371/journal.pone.0349515)
Supplement: Attachment — Submitted filename: PONE-D-24-58875_review.pdf [file pone.0349515.s001.pdf]

The prevalence of the mistreatment of older women and associated risk factors in an area of Eastern Andalusia, Spain: A cross-sectional, community-based study

All my comments are categorized as major, minor and recommendation. You may or may not consider addressing the comments that are only meant for recommendation. I hope my comments will help increase the quality of your work.

Title

<minor> Per PLoS guidelines, you should include a Short Title as Running Head.

<recommendation> Although your full title is under suggested 250 characters, I suggest keeping it under 20 words.

Abstract

Kindly note that my comments on the abstract may not be repeated in the main text, but they are intended to apply wherever relevant throughout the manuscript.

<major> The use of ageist language such as “elderly,” “aged,” “seniors” to describe older adults is highly discouraged in scientific literature. Please use other aging friendly language such as “older adults,” “older persons” throughout the manuscript. However, since “elder abuse” is a legal term, this term is accepted for its global legal implication. On the other hand, “elder” is considered somewhat accepted; however, I still discourage using this term.

<minor> While it is often stated that research on the mistreatment of older women in Spain is scarce, such claims can sometimes be overly general. Since your study is only about prevalence and associated factors, please reconfirm this assertion of research gap under this topic. And also, kindly discuss the strength, if you have not, under the “Strengths and Limitations” your study provides to fill up this research gap on older women in Spain.

<recommendation> The words like “violence,” “mistreatment,” and “abuse” may be closely related, but they are not the same concepts. Please use one of the terms that your study is primarily about and be consistent throughout the manuscript. You may describe all three terms briefly in the introduction and state which of these terms your study is specifically about. This will enhance clarity on what your study is specifically about.

<recommendation> You specified that your study is among non-institutionalized women. However, I find your conclusions for the abstract more relevant to abuse among institutionalized women, and thus you may require making it more relevant or distinct to address issues of abuse in community settings, including specific recommendations to address the issues of abuse in household settings.

Introduction

Your introduction is well-written covering major aspects of providing a background, highlighting the existing research and potential research gap. However, I have a few additional suggestions:

<major> I realized that your study is primarily about abuse in domestic settings. Please make sure you do not mix up the concepts of violence against older women with abuse against older women although these two concepts intersect; and please make sure that your writings and cited literature are primarily about abuse among older women, not about violence against women. Violence against women is another broad topic of research studied widely in Spain.

<major> International readers might not know what type of society exist in Spain. For example, is it an individualistic or collectivist society in Spain? Are families in Spain multigenerational or nuclear? Is the older care provided by family members or other informal caregivers or by assisted living professionals? These background information help readers to understand the settings related to older care in Spain.

<major> Although you discussed very briefly that there are strong regulations to protect abuse against older women, you might consider adding a separate short paragraph for global and local readers to learn what specific legal frameworks are tailored to protect older women against abuse in Spain. And, based on existing literature, I suggest you provide some justification behind higher prevalence of elder abuse among women in Spain, despite robust laws and policies against elder abuse.

<recommendation> Please add a conceptual framework to enhance clarity.

<recommendation> If you can, it is better to integrate a relevant gerontological theory to strengthen your study. Please discuss one suitable relevant gerontological theory in the introduction section and elaborate your findings in support of the existing theoretical framework.

Materials and methods

Study design, sites and participants

<major> You need to clarify the sampling approach used in your study. Was the design based on probability sampling, or did it follow a purposive or non-probability approach? The first paragraph suggests characteristics of snowball sampling, but this is not explicitly stated. On the contrary, the second paragraph suggests a random sampling. If you have used random sampling, please detail out the process. Also, please provide a rationale behind your sample size. Clear identification of the sampling method is essential for understanding the representativeness and limitations of your findings.

<minor> You have hinted a little. However, I am curious to learn whether the study was undertaken within their household or outside of the household. If it was taken in the household, what measures were taken to avoid the interruption from other family members given the sensitivity of the topic? It will be helpful to add whether or not proxies were allowed. Also, how was the data collected? Did you use paper surveys or tablet questionnaire? If paper surveys were used, how did you digitize the data. If tablet version was

used, please elaborate a little on how you designed the software and performed data management.

Measures

Geriatric Mistreatment Scale

<recommendation> Please specify how you checked the internal consistency of the Geriatric Mistreatment Scale in your study. I am asking this question because if you have used Cronbach alpha, although widely used it is not considered a robust measure to assess internal consistency. If you want to, you may consider using alternative measures such as split half or McDonald's Omega instead of using Cronbach alpha to test reliability.

Socio-demographic information

<major> The information on socio-demographic information lacks clarity. Were the variables recoded during the data analysis? Or were the variables collected in the way those were explained. If it is the former case, please make sure to detail out how the variables were recoded. Your explanation of the categories is not sufficient. For example, how did you determine whether the place was urban or rural. What standard was used? Likewise, what did "enough" vs "not enough" income level mean? These are some examples. Please add clarity to all other variables.

Statistical analyses

<minor> How was the data imported into SPSS environment? If paper-based survey was used, what were the measures taken to minimize the data-entry bias during the digitization process?

<minor> Please clarify the type of logistic regression you used to assess abuse. Is it adjusted multivariable logistic regression?

<major> Did you check for multicollinearity issues? If not, please do. Also, consider performing advanced diagnostics to check for influential observations, and conduct the treatment if necessary. Please report the value under AUROC, preferably concordance statistics for logistic regression, and then cite relevant methodological paper.

<major> Regression often washes out the variables with missing data. So, please clarify if you used any imputation process to address missing data? Or, please clarify what your final analytic sample was.

Ethical considerations

Please add a statement describing that participant had the right to refuse to response to any question, terminate the interview at any time without penalty, and were also given the option to withdraw after the interview.

Results

Maltreatment and socio-demographic/lifestyle variables

<major> Although you have several types of elder abuse enlisted, you have not defined what each of these types are under the methods section. Please, provide an operational definition to each of these subtypes, and how you measured each type and overall abuse.

<major> Please check your results for Table 2 and make sure that all of the bivariate relationships were assessed using Chi-square test, as some cell counts show potential implication of alternative method like Fisher Exact Test.

Risk factors for abuse of older women

<major> Please clarify what type of logistic regression did you use? Also, indicate your cut-off point for p-values and level of confidence intervals.

While you have mentioned using the backward elimination method, it would be helpful to briefly explain this technique for readers who may not be familiar with it. Specifically, you could describe how variables are removed based on statistical significance and how this impacts model interpretation. This description should be integrated under the methods section.

Could you also clarify whether you used a stepwise regression for backward elimination? If stepwise regression was applied, I recommend revisiting the model selection process. These techniques, although commonly used, have known limitations. They can sometimes exclude variables that may not be statistically significant but have important theoretical or practical implications. This description should be integrated under the methods section.

Consider discussing the rationale behind your modeling choices, especially in the context of your research question. Including variables with substantive relevance—even if they're not statistically significant—can enhance the interpretability and applicability of your findings. Also, kindly note that p-values are not definitive indicators of variable importance and I suggest not to solely rely on p-values. Those closer to the cut-off point of 0.05 might still provide some evidence regarding the association. Therefore, you may consider describing the variables that are not statistically significant but approach significance.

<minor> You may remove the column indicating β values in Table 3, since $OR = e^{\beta}$ in logistic regression can be easily derived.

<major> Please consider revising the interpretation for the log-odds for the first one or two results in each paragraph. This helps the reader to interpret and understand the results correctly. For example, include at least one or two interpretations in the general way logistic regressions are interpreted, including the variables you controlled . For example, after adjusting for marital status and general health, participants aged 60–69 had more than twice the odds of experiencing overall abuse compared to those aged 80 or older (OR = 2.36; 95% CI: 1.11–4.99). Similarly, those aged 70–79 had 187% higher the odds (OR = 2.87; 95% CI: 1.40–5.87) of facing overall abuse than those 80+ years after holding other variables in the model constant. Also, please report CI instead of p-values along with OR in your logistic regression interpretations. Confidence intervals are preferred more than the p-values. Please also restructure your table 3 to show what the reference category for each variable was.

Discussion

<minor> In the first paragraph, instead of reporting what sociodemographic variables were investigated, kindly report what variables were found to establish significant association with abuse.

<major> Neglect and psychological abuse are two most prevalent types of abuse. It was interesting to see such a low prevalence in your study. Kindly provide a strong justification behind such low prevalence. Also, please balance your discussion discussing both higher and lower ends of prevalence of neglect in literature. This applies to all other sections wherever applicable.

<major> To balance your discussion section, also discuss some of the variables that were close to significance, yet not significant and provide the justification in light of existing literature.

<recommendation> If you integrate a theory in the introduction section, kindly discuss your findings supporting or refuting the theory.

<recommendation> Please reduce the length of implications. You may not require three separate subheadings.
