# Supplementary material for: Prevalence and risk factors of abuse against older adult women: A cross-sectional community study in Eastern Andalusia, Spain
Source: PLoS One. 2026 May 15;21(5):e0349515. doi: 10.1371/journal.pone.0349515 (PMC13178880; doi:10.1371/journal.pone.0349515)
Supplement: Attachment — Submitted filename: Response to Reviewers.docx [file pone.0349515.s003.docx]

**RESPONSE TO THE EDITOR AND REVIEWERS**

| **Manuscript ID** | PONE-D-24-58875 |
| --- | --- |
| We would like to express our sincere appreciation to the editors and reviewers of *PLOS ONE* for their careful review and constructive feedback. We have thoroughly addressed all the comments provided, and we believe that the manuscript has been significantly strengthened as a result.  Below, we provide a detailed, point-by-point response to each of the reviewers’ suggestions and concerns. | |

**RESPONSE TO THE EDITOR'S COMMENTS**

**Additional Editor Comments:

Editor (1.1):**

**The authors should make it clear in the introduction why it's crucial to document the problem of violence and abuse against older adults. It is necessary for the authors to clarify: Even if the terms: violence," "mistreatment," and "abuse" are related in a very close way, it is necessary to deepen them conceptually and specify in the manuscript to which they refer. Consider that they are not synonymous.**

**Authors (1.1):** We appreciate the editor’s insightful observation, particularly given the complexity and cross-national variability surrounding the terminology of violence, mistreatment, and abuse in later life. Following this recommendation, we have strengthened the introduction by explicitly justifying the importance of documenting abuse against older adults’ women and by clarifying the conceptual distinctions between violence, mistreatment, and abuse. We now specify more clearly that the manuscript focuses on abuse within domestic and care-related contexts, acknowledging its conceptual boundaries while recognising the broader continuum of violence affecting older adult women. These revisions aim to enhance conceptual precision and align the terminology with current international scholarship in the field.

**Editor (1.2): For the title, there is no need to cut it to 20 words. Unify in the manuscript, where possible, the term: older adults. Emphasize how the study contributes to broadening knowledge of the phenomenon of abuse to older adults.**

**Authors (1.2):** We thank the Editor for these helpful indications. In response, we have slightly revised the title to improve its precision while maintaining clarity and coherence with the study’s aims. We have also standardised the terminology throughout the manuscript, using the term older adults consistently where appropriate. In addition, we have strengthened the Introduction and Discussion to more explicitly highlight how the study contributes to broadening current knowledge on abuse against older adults. These changes have now been incorporated into the revised manuscript.

**Editor (1.3): Clarifying in methods: the context of older adult women being studied. And, where appropriate, separate; women in their homes by type of family and women in specialized institutions for elderly care. Clarify the type of sampling performed. There are two opposing views: snowball and non-probabilistic sample. What was actually done?**

**Authors (1.3):** In response, we have clarified the study context by explicitly stating that all participants were community-dwelling older adult women living in their own homes or with family members, and that no women residing in specialised long-term care institutions were included. We have also specified the sampling approach, indicating that the study used a non-probability, purposive sampling strategy, rather than snowball sampling. These clarifications have now been incorporated into the Methods section to ensure full transparency and alignment with the study design.

**RESPONSE TO REVIEWERS’ COMMENTS**

**REVIEWER 1**

5. Review Comments to the Author

Please use the space provided to explain your answers to the questions above. You may also include additional comments for the author, including concerns about dual publication, research ethics, or publication ethics. (Please upload your review as an attachment if it exceeds 20,000 characters)

**Reviewer #1: The prevalence of the mistreatment of older women and associated risk factors in an area of Eastern Andalusia, Spain: A cross-sectional, community-based study**

**All my comments are categorized as major, minor and recommendation. You may or may not consider addressing the comments that are only meant for recommendation. I hope my comments will help increase the quality of your work.**

**Authors (1.0):** Dear reviewer, thank you very much for the time devoted to reviewing our manuscript and for your thoughtful suggestions for its improvement. All modifications made in response to your comments can be clearly viewed in the version of the manuscript with track changes enabled, as required by the journal’s editorial guidelines.

**Reviewer (1.1):**

**Title

Per PLoS guidelines, you should include a Short Title as Running Head.**

**Although your full title is under suggested 250 characters, I suggest keeping it under 20 words.**

**Authors (1.1):** Thank you for your suggestion regarding the manuscript title. Following your recommendation, the title has been revised and now contains fewer than 20 words, while preserving clarity and relevance. Additionally, a short title has been provided in accordance with the journal’s editorial guidelines.

**Reviewer (1.2):**

**Abstract

Kindly note that my comments on the abstract may not be repeated in the main text, but they are intended to apply wherever relevant throughout the manuscript.**

**The use of ageist language such as “elderly,” “aged,” “seniors” to describe older adults is highly discouraged in scientific literature. Please use other aging friendly language such as “older adults,” “older persons” throughout the manuscript. However, since “elder abuse” is a legal term, this term is accepted for its global legal implication. On the other hand, “elder” is considered somewhat accepted; however, I still discourage using this term.**

**While it is often stated that research on the mistreatment of older women in Spain is scarce, such claims can sometimes be overly general. Since your study is only about prevalence and associated factors, please reconfirm this assertion of research gap under this topic. And also, kindly discuss the strength, if you have not, under the “Strengths and Limitations” your study provides to fill up this research gap on older women in Spain.**

**The words like “violence,” “mistreatment,” and “abuse” may be closely related, but they are not the same concepts. Please use one of the terms that your study is primarily about and be consistent throughout the manuscript. You may describe all three terms briefly in the introduction and state which of these terms your study is specifically about. This will enhance clarity on what your study is specifically about.**

**You specified that your study is among non-institutionalized women. However, I find your conclusions for the abstract more relevant to abuse among institutionalized women, and thus you may require making it more relevant or distinct to address issues of abuse in community settings, including specific recommendations to address the issues of abuse in household settings.**

**Authors (1.2):** We sincerely thank the reviewer for these valuable observations. In response to the suggestions:

We have made all the requested terminological adjustments both in the abstract and consistently throughout the manuscript. Specifically, we have ensured the use of the expression “abuse against older adult women” as the central and most appropriate terminology, avoiding ageist terms such as “elderly,” “seniors,” or “aged,” and favouring people-first, aging-friendly language. The only exception is the use of “elder abuse” in reference to legal or internationally standardised terminology, as suggested.

Additionally, we have emphasised throughout the text that the study is focused exclusively on non-institutionalised older adult women, and have revised both the abstract and conclusion to make this distinction clearer. Specific references to abuse in household and community settings have been included to ensure that the conclusions and recommendations align with the actual scope and population of the study.

We have also revised the Introduction to clarify the conceptual distinctions between violence, mistreatment, and abuse, and to explain the specific focus and terminology adopted in our study to enhance conceptual clarity.

Finally, Strengths and Limitations section has been expanded to better frame the contribution of this study within the broader research context on older adult women in Spain, avoiding overgeneralisations while underscoring the study’s relevance and specificity. We modified the conclusions of the abstract to be more aligned with the findings and scope of this study.

**Reviewer (1.3):**

**Introduction

Your introduction is well-written covering major aspects of providing a background, highlighting the existing research and potential research gap.**

**However, I have a few additional suggestions:**

**I realized that your study is primarily about abuse in domestic settings. Please make sure you do not mix up the concepts of violence against older women with abuse against older women although these two concepts intersect; and please make sure that your writings and cited literature are primarily about abuse among older women, not about violence against women. Violence against women is another broad topic of research studied widely in Spain.**

**Authors (1.3):** Thank you for this observation. We have revised the introduction to make a clearer and more consistent distinction between abuse against older adult women and the broader concept of violence against women. We ensured that the definitions, terminology, and cited literature focus specifically on elder abuse in domestic settings, and we now explicitly clarify that our study adopts the narrower and more precise concept of abuse, avoiding conceptual overlap with general VAW research.

**Reviewer (1.4):**

**International readers might not know what type of society exist in Spain. For example, is it an individualistic or collectivist society in Spain? Are families in Spain multigenerational or nuclear? Is the older care provided by family members or other informal caregivers or by assisted living professionals? These background information help readers to understand the settings related to older care in Spain.

Authors (1.4):** We thank the reviewer for this valuable suggestion. In response, we have strengthened the introduction by explicitly contextualising Spain as a familistic and predominantly collectivist welfare setting, characterised by strong intergenerational ties and a care model largely sustained by family members—especially women. This information has been incorporated to clarify that elder care in Spain is primarily provided within the household, usually by informal caregivers rather than formal services, thereby offering international readers a clearer understanding of the sociocultural and care structures relevant to interpreting the study's findings.

**Reviewer (1.5):**

**Although you discussed very briefly that there are strong regulations to protect abuse against older women, you might consider adding a separate short paragraph for global and local readers to learn what specific legal frameworks are tailored to protect older women against abuse in Spain. And, based on existing literature, I suggest you provide some justification behind higher prevalence of elder abuse among women in Spain, despite robust laws and policies against elder abuse.**

**Authors (1.5):** We appreciate this valuable suggestion. In response, we have added a clearer and more explicit description of the Spanish legal context in the introduction, specifying the main regulatory frameworks relevant to the protection of older adult women—namely, Organic Law 1/2004 on gender-based violence and the provisions of the Spanish Criminal Code (Art. 173 and related articles). We also clarified that Spain lacks a specific legal definition of elder abuse, which results in fragmented protection. Additionally, we incorporated a brief explanation, supported by existing literature, discussing how the persistence of higher prevalence rates among older adult women may be related to sociocultural factors (e.g., familism, underreporting, dependency, and invisibility of psychological abuse).

**Reviewer (1.6):**

**Please add a conceptual framework to enhance clarity. If you can, it is better to integrate a relevant gerontological theory to strengthen your study. Please discuss one suitable relevant gerontological theory in the introduction section and elaborate your findings in support of the existing theoretical framework.**

**Authors (1.6):** A conceptual framework has now been explicitly incorporated in the introduction by drawing on established etiological theories of elder abuse and integrating them within an ecological perspective. This framework clarifies how individual, relational, community, and sociocultural factors jointly shape risk among older adult women. In the discussion, we have briefly elaborated on how our findings align with this ecological configuration—particularly the interplay of vulnerabilities such as loneliness, dependency, and sociocultural conditions—thereby strengthening the theoretical coherence of the manuscript.

**Reviewer (1.7):**

**Materials and methods**

**Study design, sites and participants**

**You need to clarify the sampling approach used in your study. Was the design based on probability sampling, or did it follow a purposive or non-probability approach? The first paragraph suggests characteristics of snowball sampling, but this is not explicitly stated. On the contrary, the second paragraph suggests a random sampling. If you have used random sampling, please detail out the process. Also, please provide a rationale behind your sample size. Clear identification of the sampling method is essential for understanding the representativeness and limitations of your findings.**

**Authors (1.7):** Thank you again for your observation regarding the sampling method. We have revised the section to clearly indicate that a non-probability, purposive sampling approach was used, based on the networks and dissemination capacity of collaborating institutions. Additionally, we have justified the sample size according to feasibility criteria within the project’s time frame. These clarifications have been incorporated in the revised version of the manuscript.

**Reviewer (1.8):**

**You have hinted a little. However, I am curious to learn whether the study was undertaken within their household or outside of the household. If it was taken in the household, what measures were taken to avoid the interruption from other family members given the sensitivity of the topic? It will be helpful to add whether or not proxies were allowed. Also, how was the data collected? Did you use paper surveys or tablet questionnaire? If paper surveys were used, how did you digitize the data. If tablet version was used, please elaborate a little on how you designed the software and performed data management.**

**Authors (1.8):** We appreciate this detailed and thoughtful observation, which highlights key aspects of the data collection process that are indeed essential for fully understanding the fieldwork context. Following your suggestions, we have revised the section to provide a clearer description of where the interviews were conducted, how privacy and confidentiality were ensured (particularly in household settings), and to confirm that no proxy interviews were required. We also specify that data were collected using both paper and digital formats. In most cases, responses were directly entered into the online questionnaire (Google Forms) by members of the research team. In other cases, they were first recorded on paper and later transcribed into the digital version by trained support staff. These changes have been incorporated into the section Study design, sites and participants.

**Reviewer (1.9):**

**Measures

Geriatric Mistreatment Scale**

**Please specify how you checked the internal consistency of the Geriatric Mistreatment Scale in your study. I am asking this question because if you have used Cronbach alpha, although widely used it is not considered a robust measure to assess internal consistency. If you want to, you may consider using alternative measures such as split half or McDonald’s Omega instead of using Cronbach alpha to test reliability.**

**Authors (1.9):** Thank you for your observation. In response, we have replaced the reference to Cronbach’s alpha with McDonald’s Omega, a more robust indicator of internal consistency. Specifically, the internal consistency of the Geriatric Mistreatment Scale was assessed using McDonald’s Omega, calculated in RStudio using the psych package. The results indicated an acceptable level of reliability (ω = .69; ωₕ = .55).

The manuscript has been updated accordingly in the “Measures” section.

**Reviewer (1.10):**

**Socio-demographic information**

**The information on socio-demographic information lacks clarity. Were the variables recoded during the data analysis? Or were the variables collected in the way those were explained. If it is the former case, please make sure to detail out how the variables were recoded. Your explanation of the categories is not sufficient. For example, how did you determine whether the place was urban or rural. What standard was used? Likewise, what did “enough” vs “not enough” income level mean? These are some examples. Please add clarity to all other variables.**

**Authors (1.10):** Thank you for this helpful observation. Several socio-demographic variables were originally collected in multiple categories and later recoded to improve clarity and statistical power. We have now specified which variables were recoded, the criteria used (e.g., rural defined as <10,000 inhabitants; income recoded using the national IPREM benchmark), and clarified the content of each category (e.g., the “low” education group includes basic literacy and primary education). These revisions have been incorporated into the “Socio-demographic information” section to enhance transparency.

**Reviewer (1.11):**

**Statistical analyses**

**How was the data imported into SPSS environment? If paper-based survey was used, what were the measures taken to minimize the data-entry bias during the digitization process?**

**Authors (1.11):** We appreciate this observation. In response, we have introduced the following clarification in the manuscript:

*Survey responses were exported from the digital form as a CSV file and imported into SPSS. For participants who completed the questionnaire on paper, trained research assistants manually entered the responses into the digital database. To minimise data-entry bias, a double-entry verification protocol was applied, and random checks were conducted on 10% of the entries.*

**Reviewer (1.12):**

**Please clarify the type of logistic regression you used to assess abuse. Is it adjusted multivariable logistic regression?**

**Authors (1.12):** Dear reviewer, we appreciate this comment. We confirm that we performed an adjusted multivariable logistic regression analysis, including all relevant socio-demographic and lifestyle variables simultaneously in the model. We have now specified this in the "Statistical analyses" section to enhance clarity.

**Reviewer (1.13):**

**Did you check for multicollinearity issues? If not, please do. Also, consider performing advanced diagnostics to check for influential observations, and conduct the treatment if necessary. Please report the value under AUROC, preferably concordance statistics for logistic regression, and then cite relevant methodological paper.**

**Authors (1.13):** Thank you for your insightful comment. In response, we confirm that multicollinearity was assessed prior to model estimation. Specifically, tolerance and variance inflation factor (VIF) diagnostics were computed for all predictors in the logistic regression models. All VIF values ranged between 1.03 and 1.40, and tolerance values ranged from 0.71 to 0.97—both well within accepted thresholds (VIF < 5; Tolerance > 0.2), indicating no multicollinearity concerns.

We also conducted an evaluation of influential observations using Cook’s Distance. None of the cases exceeded the commonly accepted threshold of D > 1, suggesting no undue influence of individual data points on model estimates; therefore, all cases were retained in the final models.

Regarding model performance, we evaluated the discriminative capacity of the logistic regression models by calculating the Area Under the Receiver Operating Characteristic Curve (AUROC). Concordance statistics (c-statistics) were calculated for each model. The AUROC values ranged from 0.67 (neglect) to 0.82 (any form of abuse), indicating fair to good discriminative power according to standard interpretation criteria (Mandrekar, 2010). These procedures have been described in the Methods section (Statistical Analyses), and appropriate references have been included.

**Reviewer (1.14):**

**Regression often washes out the variables with missing data. So, please clarify if you used any imputation process to address missing data? Or, please clarify what your final analytic sample was.**

**Authors (1.14):** We again thank the reviewer for this important observation. In our study, no cases were lost due to missing data for the variables included in the logistic regression models. As we were able to verify in the case processing summaries provided by SPSS for each model, the total number of participants analysed was 209, with no cases excluded due to missing values. Therefore, imputation techniques were not applied, and all analyses were performed with the full sample (N = 209). We have made the following final clarification in the statistical analysis section of the manuscript:

*"No missing data were observed for the variables included in the regression models; therefore, the full sample of 209 participants was retained in all analyses."*

**Reviewer (1.15):**

**Ethical considerations**

**Please add a statement describing that participant had the right to refuse to response to any question, terminate the interview at any time without penalty, and were also given the option to withdraw after the interview.**

**Authors (1.15):** Dear reviewer, we have revised the Ethical Considerations section to clarify participants’ rights during and after the interview. In particular, we have added a sentence specifying that participants were informed they could refuse to answer any question, terminate the interview at any time without penalty, and withdraw their participation after the interview if they so desired.

**Reviewer (1.16):**

**Results

Maltreatment and socio-demographic/lifestyle variables**

**Although you have several types of elder abuse enlisted, you have not defined what each of these types are under the methods section. Please, provide an operational definition to each of these subtypes, and how you measured each type and overall abuse.**

**Authors (1.16):** As requested, we have now clarified the operational definitions of each subtype of elder abuse (physical, psychological, economic, sexual and neglect) in the “Measures” section, under the “Geriatric Mistreatment Scale” subsection. We also specified how each type of abuse was computed based on item grouping and dichotomous coding, and how overall abuse was derived from the 22-item scale.

**Reviewer (1.17):**

**Please check your results for Table 2 and make sure that all of the bivariate relationships were assessed using Chi-square test, as some cell counts show potential implication of alternative method like Fisher Exact Test.**

**Authors (1.17):** Authors (1.14): We thank the reviewer for this important observation, which we had overlooked but did make. In our bivariate analyses (Table 2), Pearson's chi-squared test was used to assess associations. However, in cases where the assumptions of the chi-squared test were not met (i.e., more than 20% of the cells had expected counts <5), Fisher's exact test was applied, as recommended. This clarification has been added to the Statistical Analysis section of the revised manuscript.

**Reviewer (1.18):**

**Risk factors for abuse of older women**

**Please clarify what type of logistic regression did you use? Also, indicate your cut-off point for p-values and level of confidence intervals.**

**While you have mentioned using the backward elimination method, it would be helpful to briefly explain this technique for readers who may not be familiar with it. Specifically, you could describe how variables are removed based on statistical significance and how this impacts model interpretation. This description should be integrated under the methods section.**

**Could you also clarify whether you used a stepwise regression for backward elimination? If stepwise regression was applied, I recommend revisiting the model selection process. These techniques, although commonly used, have known limitations. They can sometimes exclude variables that may not be statistically significant but have important theoretical or practical implications. This description should be integrated under the methods section.**

**Authors (1.18):** We appreciate the reviewer’s insightful comments. As requested, we have now specified in the Statistical Analyses section that we conducted an adjusted multivariable binary logistic regression analysis using a backward stepwise method based on the likelihood ratio criterion, with a significance cut-off of *p* > 0.05 for variable removal and a confidence level of 95%. Additionally, we briefly explained the rationale for using this approach, noting that variables were sequentially removed based on statistical insignificance in order to retain the most parsimonious model while minimising overfitting. We also acknowledged that although stepwise methods are commonly used, they have limitations, as they may exclude variables with important theoretical or practical relevance despite lacking statistical significance. This limitation is now explicitly discussed in the Limitations section to promote transparent interpretation of our findings. You may find the revised paragraph at “Statistical analyses” subsection.

**Reviewer (1.19):**

**Consider discussing the rationale behind your modeling choices, especially in the context of your research question. Including variables with substantive relevance—even if they're not statistically significant—can enhance the interpretability and applicability of your findings. Also, kindly note that p-values are not definitive indicators of variable importance and I suggest not to solely rely on p-values. Those closer to the cut-off point of 0.05 might still provide some evidence regarding the association. Therefore, you may consider describing the variables that are not statistically significant but approach significance.**

**Authors (1.19):** We thank the reviewer for this insightful comment. Following the recommendation, we incorporated a brief discussion of variables that, although not statistically significant, showed p-values close to the conventional 0.05 threshold. These near-threshold patterns (e.g., age for neglect, housing for sexual abuse, education for economic abuse, dependency and social services use for several abuse types) were now described in the Results section to enhance interpretability and acknowledge their potential substantive relevance.

**Reviewer (1.20):**

**You may remove the column indicating β values in Table 3, since OR=e^β in logistic regression can be easily derived.**

**Authors (1.20):** Thank you for the observation, we have removed the corresponding column from the table.

**Reviewer (1.21):**

**Please consider revising the interpretation for the log-odds for the first one or two results in each paragraph. This helps the reader to interpret and understand the results correctly. For example, include at least one or two interpretations in the general way logistic regressions are interpreted, including the variables you controlled . For example, after adjusting for marital status and general health, participants aged 60–69 had more than twice the odds of experiencing overall abuse compared to those aged 80 or older (OR = 2.36; 95% CI: 1.11–4.99). Similarly, those aged 70–79 had 187% higher the odds (OR = 2.87; 95% CI: 1.40–5.87) of facing overall abuse than those 80+ years after holding other variables in the model constant. Also, please report CI instead of p-values along with OR in your logistic regression interpretations. Confidence intervals are preferred more than the p-values. Please also restructure your table 3 to show what the reference category for each variable was.**

**Authors (1.21):** We appreciate this helpful suggestion. Following your recommendation, we revised the presentation of the logistic regression results to include clear and standard interpretations of the odds ratios for the first one or two findings in each subsection. These interpretations now explicitly indicate that the estimates are adjusted for all variables included in the model and rely on OR and 95% confidence intervals rather than p-values, as preferred in logistic regression reporting.

We have also ensured that the reference categories for all variables are clearly identifiable. As noted in Table 3, the reference groups were already specified in the original submission and remain unchanged in the revised manuscript: Age range (80 or older), Marital status (married), Dependency situation (yes), Feeling of loneliness (no), Use of social services in the last month (no), and General health (satisfied).

**Reviewer (1.22):**

**Discussion

In the first paragraph, instead of reporting what sociodemographic variables were investigated, kindly report what variables were found to establish significant association with abuse.**

**Authors (1.22):** Following the reviewer’s recommendation, a paragraph has been added at the beginning of the discussion summarizing the associations between all forms of abuse and the sociodemographic variables, as well as those corresponding to each specific type of abuse.

**Reviewer (1.23):**

**Neglect and psychological abuse are two most prevalent types of abuse. It was interesting to see such a low prevalence in your study. Kindly provide a strong justification behind such low prevalence. Also, please balance your discussion discussing both higher and lower ends of prevalence of neglect in literature. This applies to all other sections wherever applicable.**

**Authors (1.23):** Thank you for this insightful reflection, which enriches our discussion. In the case of psychological violence, our study aligns with previous literature in showing that psychological abuse is the most prevalent. However, it diverges from prior findings regarding neglect. In this regard, we have included in the discussion a potential explanatory factor for this observation, referring to the type of care provision offered by Spanish society, and specifically in Andalusia.

**Reviewer (1.24):**

**To balance your discussion section, also discuss some of the variables that were close to significance, yet not significant and provide the justification in light of existing literature.

Authors (1.24):** We appreciate the reviewer’s comment. We have added brief reflections in the Discussion addressing variables that were close to significance, contextualising these trends in light of existing literature.

**Reviewer (1.25):**

**If you integrate a theory in the introduction section, kindly discuss your findings supporting or refuting the theory.**

**Authors (1.25):** Thank you for this valuable suggestion. We have now explicitly linked our findings to the theoretical framework introduced in the manuscript. In the Discussion section, we incorporated a statement clarifying how the results support an ecological and critical-ecological interpretation of elder abuse, highlighting the interplay between individual, relational and sociocultural factors. We trust that this addition addresses the reviewer’s comment.

**Reviewer (1.26):**

**Please reduce the length of implications. You may not require three separate subheadings.**

**Authors (1.26):** Thank you for the comment. The implications section has now been shortened as requested. We have also consolidated the content into fewer subheadings to improve clarity and coherence.

**Final Comment to Reviewer 1**

We would like to express our sincere appreciation to Reviewer 1 for the thorough and constructive evaluation of our manuscript. Your insightful observations and detailed suggestions substantially contributed to refining the clarity, analytical depth, and overall quality of the study. We have carefully addressed each point raised and integrated the recommended improvements with the greatest diligence. We hope that the revised version reflects the value of your feedback and meets the expectations conveyed in your review. Thank you once again for the time and expertise dedicated to strengthening our work.

**REVIEWER 2**

**Reviewer (2.0):**

**Reviewer #2: I would like to express my sincere gratitude to the editor for the opportunity to review the study titled "The prevalence of the mistreatment of older women and associated risk factors in an area of Eastern Andalusia, Spain: A cross-sectional, community-based study." I appreciate the effort into this research and look forward to providing constructive feedback to enhance its quality and impact.**

**Authors (2.0):** Dear reviewer, thank you very much for the time devoted to reviewing our manuscript and for your thoughtful suggestions for its improvement. All modifications made in response to your comments can be clearly viewed in the version of the manuscript with track changes enabled, as required by the journal’s editorial guidelines.

**Reviewer (2.1):**

**I have outlined several comments and suggestions below that aim to strengthen the overall quality and clarity of the manuscript:1. Some parts are overly verbose. Aim for more concise sentences to maintain reader engagement.**

**Authors (2.1):** We appreciate this observation. In response, we have revised several sections of the manuscript to improve clarity and conciseness, particularly in the Introduction, Methods, and Discussion. Redundant phrases have been removed, long sentences have been streamlined, and wording has been adjusted to enhance readability while preserving the scientific meaning.

**Reviewer (2.2):**

**2. The literature review could be strengthened by including more recent studies or contrasting findings to highlight gaps in existing research.**

**Authors (2.2):** With gratitude for your contribution, the authors have reviewed the most recent papers published on the topic and have incorporated these findings into both the introduction and the discussion sections.

**Reviewer (2.3):**

**3. The sample size (209 participants) may limit the generalizability of the findings. Write about sample size.**

**Authors (2.3):** Thank you for the suggestion. We have now explicitly highlighted the limitations associated with the sample size and provided a more detailed explanation of the sampling approach in the corresponding subsection of the manuscript.

**Reviewer (2.4):**

**4. More detail on the Geriatric Maltreatment Scale's validation process would enhance credibility. How was it adapted for this study?**

**Authors (2.4):** In response, we have expanded the description of the Geriatric Mistreatment Scale in the Measures section, incorporating additional information on its validation process. We have also detailed the specific steps taken in this study, including a brief content review for contextual suitability and a pilot test with 11 older adult women, which confirmed that the items were clearly understood and culturally appropriate. These revisions have been made to strengthen the instrument’s credibility and respond to your comment, as well as to align with related feedback from other reviewers.

**Reviewer (2.5):**

**5. Some claims lack sufficient backing from the data presented. Ensure that all assertions are supported by the results in discussion.**

**Authors (2.5):** Thank you for the suggestion. An in-depth review and refinement of the results and discussion section has been carried out, ensuring a clear and coherent connection between the findings and their subsequent interpretation. In this regard, we consider that in the present version of the manuscript, all the assertions are supported by the results in discussion.

**Reviewer (2.6):**

**6. The implications for practice and policy could be more explicitly stated. What specific actions should be taken based on the findings?**

**Authors (2.6):** Based on the valuable suggestion provided by the reviewer, specific actions to be undertaken have been defined based on the findings, from both practical and policy-oriented perspectives.

**Reviewer (2.7):**

**7. The discussion of limitations could be expanded. Consider discussing the potential impact of cultural factors on the findings.**

**Authors (2.7):** Thank you for the recommendation. An expanded version of the limitations is presented, which additionally incorporates the impact of sociocultural variables that should be considered when interpreting the findings. New limitations have also been incorporated, particularly in relation to the methodology employed (backward stepwise logistic regression).

**Reviewer (2.8):**

**8. It could reiterate the importance of addressing the identified issues in a more impactful manner.**

**Authors (2.8):** Following this suggestion, the wording has been revised in order to achieve a greater emphasis on the need to ensure the applicability of the findings so as to optimize the impact of the empirical evidence.

**Reviewer (2.9):**

**9. Suggest future research directions more explicitly to guide subsequent studies.**

**Authors (2.9):** We thank the reviewer for his/her confidence in our ability to identify future research. The proposed future research directions have been specified in greater detail, and their relevance has been thoroughly justified.

**Reviewer (2.10):**

**10. The manuscript is generally well-written, but proofreading for grammatical errors and clarity is recommended.**

**Authors (2.10):** Thank you for this suggestion. We have carefully proofread the entire manuscript to address minor grammatical issues, improve clarity and enhance fluency. Several sentences were revised for readability, and terminology was standardised throughout the text. We have also incorporated an additional language review by a native English speaker to ensure the manuscript meets the highest standards of clarity and academic writing.

**Final comment for editors and reviewers:**

We would like to express our sincere gratitude to the editors and reviewers for the time, effort, and careful consideration invested in evaluating our manuscript. We greatly appreciate the depth and precision of the feedback provided, which has undoubtedly strengthened the quality, clarity, and methodological robustness of the study. Throughout the revision process, we have addressed all comments with the highest diligence and scientific rigor. Where necessary, we consulted specialists in statistical analysis to ensure that the methodological revisions were conducted accurately and in line with best practices. In addition, the entire revised manuscript has been reviewed by a native English speaker to improve clarity and fluency. We sincerely hope that the modifications implemented are aligned with the expectations of the reviewers and the editorial team, and that the revised version meets the standards of excellence required by the journal. We thank you again for your constructive contributions and for the opportunity to improve our work.

Reviewers' comments:

Reviewer's Responses to Questions

**Comments to the Author**

1. Is the manuscript technically sound, and do the data support the conclusions?

The manuscript must describe a technically sound piece of scientific research with data that supports the conclusions. Experiments must have been conducted rigorously, with appropriate controls, replication, and sample sizes. The conclusions must be drawn appropriately based on the data presented.

Reviewer #1: Partly

Reviewer #2: Yes

2. Has the statistical analysis been performed appropriately and rigorously?

Reviewer #1: Yes

Reviewer #2: Yes

3. Have the authors made all data underlying the findings in their manuscript fully available?

The [PLOS Data policy](http://www.plosone.org/static/policies.action#sharing) requires authors to make all data underlying the findings described in their manuscript fully available without restriction, with rare exception (please refer to the Data Availability Statement in the manuscript PDF file). The data should be provided as part of the manuscript or its supporting information, or deposited to a public repository. For example, in addition to summary statistics, the data points behind means, medians and variance measures should be available. If there are restrictions on publicly sharing data—e.g. participant privacy or use of data from a third party—those must be specified.

Reviewer #1: Yes

Reviewer #2: Yes

4. Is the manuscript presented in an intelligible fashion and written in standard English?

PLOS ONE does not copyedit accepted manuscripts, so the language in submitted articles must be clear, correct, and unambiguous. Any typographical or grammatical errors should be corrected at revision, so please note any specific errors here.

Reviewer #1: Yes

Reviewer #2: Yes
