# Supplementary material for: Prevalence and risk factors of abuse against older adult women: A cross-sectional community study in Eastern Andalusia, Spain
Source: PLoS One. 2026 May 15;21(5):e0349515. doi: 10.1371/journal.pone.0349515 (PMC13178880; doi:10.1371/journal.pone.0349515)
Supplement: Attachment — Submitted filename: Response_to_Reviewers_auresp_2.docx [file pone.0349515.s004.docx]

**RESPONSE LETTER TO EDITORS AND REVIEWERS**

| **Manuscript ID** | PONE-D-24-58875R1 |
| --- | --- |
| **Title** | Prevalence and risk factors of abuse against older adult women: a cross-sectional community study in Eastern Andalusia, Spain |
| **General Comment for Editors and Reviewers** | We would like to express our sincere appreciation to the editors and reviewers of *PLOS ONE* for their careful review and constructive feedback. We have thoroughly addressed all the comments provided, and we believe that the manuscript has been significantly strengthened as a result.    Below, we provide a detailed, point-by-point response to each of the reviewers’ suggestions and concerns. |

**Additional Editor Comments:**

**We acknowledge author efforts to address the reviwers comments, however, some methodological and interpretative issues require clarification, particularly regarding sampling, measurement reliability and interpretation of the high prevalence estimate.**

**Editor’s Comment 1. The study used non-probability purposive sampling through institutional networks (women’s associations and social services). This approach may introduce selection bias, potentially affecting prevalence estimates. Thus, author must clarify the recruitment process in “more detail” and explicitly acknowledge the limitations for generalizability in the discussion section.**

**Authors’ Response 1.**

We thank the Editor for this comment. We have expanded the description of the recruitment process in the “Materials and Methods” section to provide greater detail regarding the institutional networks involved, how contact with potential participants was facilitated, and the protective measures taken prior to data collection. Specifically, we now clarify that participants were recruited through women’s associations, neighbourhood organisations, and municipal social services (via the home help service provider), and that collaborating institutions received an advance letter presenting the research team and the purpose of the study.

In addition, we have explicitly acknowledged the limitations of non-probability purposive sampling in the Limitations section of the Discussion, noting that this approach may introduce selection bias and that the resulting estimates should not be generalised to the broader population of older adult women in Spain or beyond. These changes are marked in the revised manuscript.

**Editor’s Comment 2. The reported prevalence of 49.3% abuse within 12 months is considerably higher than most international estimates. The study classifies a participant as abused if she answered “yes” to at least one of the 22 items in the Geriatric Mistreatment Scale, and this approach inflates prevalence estimates. Thus, it becomes extremely vital to provide a stronger explanation in the discussion, noting that the estimate may reflect a) the screening nature of the instrument, b) classification of abuse based on any positive item out of 22 , c) and non-probability community sampling.**

**Authors’ Response 2.**

We fully agree with the Editor’s observation. We have strengthened the explanation of the high prevalence estimate in the Discussion section. The revised text now explicitly acknowledges that the figure of 49.3% should be interpreted in light of three methodological characteristics: (a) the GMS functions as a screening instrument designed to identify the possible presence of abuse, rather than to produce clinically confirmed case rates; (b) the classification criterion—any affirmative response to at least one of the 22 items—is broad by design and likely captures sub-threshold or borderline experiences that narrower instruments would not detect; and (c) the non-probability purposive sampling may have recruited women with greater exposure or awareness of abuse situations.

We have also added a direct comparison with other studies that used similar screening approaches and reported comparable or higher prevalence rates, contextualising our findings within the broader literature. These changes are clearly marked in the revised manuscript.

**Editor’s Comment 3. Internal consistency for the Geriatric Mistreatment Scale in this sample was moderate (ω ≈ 0.69). Author must report reliability estimates for each abuse subtype or provide additional justification for using the combined “any abuse” variable in the analysis.**

**Authors’ Response 3.**

We thank the Editor for this important methodological comment. We have addressed it through two complementary strategies.

First, regarding reliability estimates at the subtype level: the dataset available for analysis contains binary indicators reflecting whether each participant experienced a given subtype of abuse (yes/no), rather than item-level responses for each of the 22 individual items of the GMS. Because internal consistency indices such as McDonald’s omega or KR-20 require item-level data (i.e., the response to each individual item within a subtype), it is not possible to compute these statistics for each subtype directly from the available data. We have acknowledged this explicitly in the revised Measures section.

Second, to provide the additional justification requested for using the combined “any abuse” variable, we have added the following evidence-based arguments in the Measures section and in the Limitations section:

a) Convergent validity of the subtypes: The phi (φ) correlations between abuse subtypes—computed from the present dataset—demonstrate meaningful inter-subtype associations, particularly among psychological, economic, sexual, and neglect dimensions (e.g., φ = 0.47 for neglect–sexual; φ = 0.37 for psychological–sexual; φ = 0.33 for psychological–financial), consistent with the polyvictimisation literature on elder abuse. These associations support the conceptual coherence of a combined abuse indicator.
b) High co-occurrence of abuse forms: Of the 103 women who experienced any form of abuse (49.3%), 55.3% experienced two or more forms simultaneously (45.6% of all 103 abused women experienced multiple forms). This polyvictimisation pattern justifies the use of a composite “any abuse” variable as the primary outcome in logistic regression, since distinct subtypes frequently co-occur and are unlikely to operate as fully independent constructs.
c) Consistency with the GMS validation study and prior usage: The GMS was validated and has been used in prior research with the combined score as the primary outcome variable (Giraldo-Rodríguez & Rosas-Carrasco, 2013; Piña-Escudero et al., 2021; Vilar-Compte et al., 2018). The moderate omega (ω = 0.69) is consistent with what is expected for a heterogeneous multidimensional construct, in which abuse subtypes are conceptually related but not redundant.
d) Recognised limitation: We have added an explicit acknowledgement in the Limitations section that the moderate internal consistency of the scale and the impossibility of computing subtype-level reliability from the available data represent a methodological limitation, and we recommend that future studies collect item-level data to enable more granular psychometric analyses.

These additions are clearly marked in the revised manuscript.

**Editor’s Comment 4. The manuscript used backward stepwise logistic regression, which may lead to unstable models or exclusion of theoretically important variables. Thus, author must briefly justify the modeling strategy and discuss its limitations in the limitations section.**

**Authors’ Response 4.**

We appreciate this observation. We have added a brief methodological justification for the use of backward stepwise logistic regression in the “Statistical Analysis” subsection. The text now clarifies that this approach was chosen in an exploratory context, given the lack of a fully established theoretical model for selecting risk factors specific to older adult women in Southern Spain, and that the method allows the data to inform variable selection while retaining parsimony. We acknowledge that this approach may be sensitive to sample characteristics and could lead to the exclusion of theoretically relevant variables not supported by the specific sample.

In addition, we have expanded the Limitations section to explicitly discuss the limitations of backward stepwise regression, including the risk of model instability, potential overfitting in small-to-moderate samples, and the possibility that variables of theoretical importance may be excluded if their individual contribution does not reach the retention threshold. We recommend that future studies with larger samples consider block-entry or theory-driven regression strategies. These changes are marked in the revised manuscript.

**Editor’s Comment 5. Ensure consistent use of the term “abuse against older adult women” throughout the manuscript.**

**Authors’ Response 5.**

We thank the Editor for this comment. We have conducted a thorough review of the entire manuscript to standardise the use of the term “abuse against older adult women” where appropriate. Instances in which alternative formulations appeared (e.g., “elder abuse,” “old-age abuse,” or “mistreatment of older women”) have been revised for consistency, except in direct references to specific validated instruments (such as the “Geriatric Mistreatment Scale”) or in citations of prior literature where the original terminology is maintained. These changes are marked in the revised manuscript.

**Editor’s Comment 6. Several socio-demographic variables (such as age, education, and income) were recoded into broad categories, which may reduce variability & statistical precision. The manuscript should briefly justify these recoding decisions and explain how the chosen categories align with previous research or contextual considerations.**

**Authors’ Response 6.**

We agree that a justification for the recoding decisions was missing. We have added a paragraph in the “Socio-demographic information” subsection explaining that age, education, and income were grouped into broad categories to ensure sufficient cell sizes for statistical analysis (given a total sample of *N* = 209), to align with the categorisations used in comparable studies of older adult abuse in Spain and internationally, and to reflect contextually meaningful thresholds. For example, the binary age split at 80 years reflects the WHO classification of “old-old” adults and is widely used in gerontological research. The income categorisation is based on the IPREM (Indicador Público de Renta de Efectos Múltiples) threshold used in Spanish social policy. These additions are marked in the revised manuscript.

**Editor’s Comment 7. Author may do minor language editing to remove repetitions.**

**Authors’ Response 7.**

We have carefully reviewed the full manuscript to identify and eliminate repetitive passages. Redundant sentences and phrases—particularly in the Introduction and Discussion sections—have been revised or removed. The manuscript now reads more concisely, with no substantive content loss. All changes are marked in the revised version.

**Reviewer #1:**

**I appreciate how the authors worked to improve the manuscript and address/integrate my comments/suggestions. Thank you for your sincere effort. I do notice that the internal reliability of the Geriatric Mistreatment Scale is not that great; but it is what it is based on the dataset you analyzed. If possible, please acknowledge this limitation in your manuscript and if you are able to justify briefly why you think your McDonald's omega value was only acceptable. Other than that, your revised version now has robust methods, clear presentation of results, and succint overview and discussion.**

**Authors’ Response 1.**

We sincerely thank Reviewer 1 for their positive assessment of the revised manuscript and for their constructive observation regarding the internal reliability of the GMS. We have now explicitly acknowledged the moderate omega value (ω = 0.69) as a limitation in the Limitations section. In addition, we offer the following brief justification for why this value, while not optimal, is interpretively acceptable in the context of this study:

The GMS is a multidimensional scale covering five conceptually distinct types of abuse (physical, psychological, economic, sexual, and neglect). Unlike unidimensional scales, in which all items are expected to measure the same underlying construct and high internal consistency is a primary criterion, multidimensional instruments capturing heterogeneous constructs typically yield lower omega values. A moderate omega is expected—and is not necessarily a validity concern—when the scale is designed to screen across conceptually distinct subtypes rather than to measure a single latent trait. This is consistent with the original validation study of the GMS (Giraldo-Rodríguez & Rosas-Carrasco, 2013), and with the multidimensional structure of elder abuse more broadly.

Furthermore, the moderate omega in this sample may also reflect the relatively low prevalence of certain subtypes (e.g., neglect: 7.7%), which constrains the variance available for inter-item covariation and can systematically reduce omega estimates. These considerations have been added to the Limitations section of the revised manuscript.

**Final Comment for Editors and Reviewers**

*We would like to express our sincere gratitude to the reviewers and the editors of PLOS ONE for their thoughtful feedback and constructive comments. We sincerely hope that the revisions undertaken adequately address the concerns raised and that the revised manuscript meets the standards of quality expected by the journal.*
